# The influential factors and non-pharmacological interventions of cognitive impairment in children with ischemic stroke

**DOI:** 10.3389/fneur.2022.1072388

**Published:** 2022-12-15

**Authors:** Gang Xu, Fuchun Hao, Weiwei Zhao, Jiwen Qiu, Peng Zhao, Qian Zhang

**Affiliations:** ^1^Rehabilitation Branch, Tianjin Children's Hospital/Tianjin University Children's Hospital, Tianjin, China; ^2^Medicine & Nursing Faculty, Tianjin Medical College, Tianjin, China; ^3^Chinese Teaching and Research Section, Tianjin Beichen Experimental Middle School, Tianjin, China; ^4^Research Center of Experimental Acupuncture Science, Tianjin University of Traditional Chinese Medicine, Tianjin, China; ^5^School of Medical Technology, Tianjin University of Traditional Chinese Medicine, Tianjin, China; ^6^Child Health Care Department, Tianjin Beichen Women and Children Health Center, Tianjin, China

**Keywords:** pediatric, ischemic stroke, cognitive impairment, intervention, non-pharmacological

## Abstract

**Background:**

The prevalence of pediatric ischemic stroke rose by 35% between 1990 and 2013. Affected patients can experience the gradual onset of cognitive impairment in the form of impaired language, memory, intelligence, attention, and processing speed, which affect 20–50% of these patients. Only few evidence-based treatments are available due to significant heterogeneity in age, pathological characteristics, and the combined epilepsy status of the affected children.

**Methods:**

We searched the literature published by Web of Science, Scopus, and PubMed, which researched non-pharmacological rehabilitation interventions for cognitive impairment following pediatric ischemic stroke. The search period is from the establishment of the database to January 2022.

**Results:**

The incidence of such impairment is influenced by patient age, pathological characteristics, combined epilepsy status, and environmental factors. Non-pharmacological treatments for cognitive impairment that have been explored to date mainly include exercise training, psychological intervention, neuromodulation strategies, computer-assisted cognitive training, brain-computer interfaces (BCI), virtual reality, music therapy, and acupuncture. In childhood stroke, the only interventions that can be retrieved are psychological intervention and neuromodulation strategies.

**Conclusion:**

However, evidence regarding the efficacy of these interventions is relatively weak. In future studies, the active application of a variety of interventions to improve pediatric cognitive function will be necessary, and neuroimaging and electrophysiological measurement techniques will be of great value in this context. Larger multi-center prospective longitudinal studies are also required to offer more accurate evidence-based guidance for the treatment of patients with pediatric stroke.

## 1. Introduction

Childhood arterial ischemic stroke is defined as a cerebrovascular event that occurs between 1 month and 18 years of age. Arterial ischemic stroke is characterized by an acute-onset neurological deficit due to an infarct in an arterial territory consistent with the clinical syndrome (1). The annual incidence of stroke in children is 1.3–13/100,000 (2), and it has risen by 35% between 1990 and 2013 (3). The prognosis for post-stroke recovery in affected children is no better than that of patients with adult stroke (3). The primary difference between these two patient populations is that in adults, a stroke can result in a loss of functional independence, whereas in children, it can also interfere with their growth and development due to prolonged neurological dysfunction (4–7). Children, young adults, and their parents exhibit high levels of unmet needs across a range of health domains in the months and years after pediatric stroke (8). Due to delays in presentation, only about 2% of children are eligible for treatment with thrombolysis and thrombectomy (9). Therefore, rehabilitation therapies for pediatric stroke are important, which improve outcomes after hyperacute treatment (10).

Approximately 20–50% of patients with pediatric ischemic stroke exhibit signs of cognitive impairment (3), affecting both executive function and behavioral traits, including intelligence, memory, attention, and processing speed. The intelligence quotient (IQ) values of patients with pediatric ischemic stroke are generally reported to be on the lower end of the normal range while being significantly lower on average than those in healthy age-matched populations (11–13). Notably, patients exhibit significantly more damage to operational IQ values relative to speech IQ values (12). In one study of the neuropsychological characteristics of 49 children after ischemic stroke, the average performance of these children in attention and executive function tasks was significantly lower than that of the healthy control children, with 67% of the children exhibiting impairment when completing attention tasks and 30% of the children exhibiting impaired executive function (13). Processing speed (12, 14, 15) and working memory (4, 14) are also significantly impaired in patients with ischemic stroke.

Compared with the adult stroke population, there is conflicting evidence about whether children's prognosis is more favorable and whether children recover better after stroke than adults (10, 16, 17). The plasticity and the selective vulnerability are widely held assumptions (18). Such as, children with ischemic stroke have different cognitive prognoses depending on their age groups (19, 20). Two mechanisms of recovery after nerve injury have been proposed, namely, behavioral recovery and compensation. For the rehabilitation of children with stroke, the influence of natural development should also be considered. The immature brain, however, is a dynamic environment with significant changes to the cellular composition, neural circuitry, and blood flow occurring throughout childhood (21).

Developing a better understanding of the risk of cognitive impairment and other adverse outcomes in children following ischemic stroke occurrence is critically important to parents, clinicians, and patients (22). Relatively few studies have been conducted on cognitive impairment in children after ischemic stroke. Therefore, the present review explores the factors associated with the prognosis of these patients and discusses treatment strategies aimed at alleviating post-ischemic stroke cognitive impairment to provide a foundation for future interventional and patient management strategies.

## 2. Methods

We searched the literature published by Web of Science, Scopus, and PubMed, which researched non-pharmacological rehabilitation interventions for cognitive impairment following pediatric ischemic stroke. Keywords are as follows: pediatric, ischemic stroke, cognitive, and intervention. The search period is from the establishment of the database to January 2022.

## 3. Factors affecting cognitive impairment

### 3.1. Demographic characteristics: Chronological age

Some reports suggested that stroke onset before the age of 1 year is associated with poorer cognitive outcomes (4, 19), while other reports have found poorer outcomes tend to occur in patients below the age of 1 year and over the age of 6 years (20), with children between these two ages having better outcomes on average (19). Still, other studies suggested that children between the ages of 5 and 10 years have the best prognosis after stroke, with children outside of this age range exhibiting a poorer prognosis (11). These studies all seem to agree that stroke outcomes are poor in children under the age of 1 year and over the age of 10 years. One study reported significantly different neuropsychological outcomes when comparing strokes occurring during the perinatal period to those occurring in children (29 days−18 years old) (23). Overall, these results suggest that age is a key factor in the cognitive outcome of children after a stroke.

### 3.2. Stroke features

#### 3.2.1. Lesion characteristics

Larger infarct area (20), larger lesion volume (4, 12), and simultaneous cortical and subcortical involvement (4) are all associated with poorer cognitive outcomes. One study found that Language and verbal IQ scores were significantly lower (*p* < 0.01) among patients with lesions in the left hemisphere as opposed to the right in 184 children retrospectively (24). In contrast, other studies have detected no differences in cognitive outcomes as a function of lesion laterality (14, 23). Kornfeld et al. suggested that children experience a significant reduction in their resting-state functional connection of the bilateral parietal lobes following stroke incidence while also exhibiting positively correlated reductions in processing speed and perceptual reasoning relative to healthy controls (25). Overall, these data clearly emphasize the relationship between pathological lesion characteristics and prognosis.

#### 3.2.2. Comorbidity (epilepsy)

Many children with arterial ischemic stroke present with acute symptomatic seizures, and survivors frequently develop remote symptomatic seizures and epilepsy. Remote symptomatic seizures were defined as any seizure occurring ≥30 days after stroke ictus. Definite epilepsy was defined as ≥2 unprovoked seizures occurring ≥24 h apart (26). According to the literature, the manifestation of epilepsy after pediatric stroke varies between 13 and 67%, depending on the study population (27). The risk factors included early seizures, young age, cortical lesions, and multiple infarctions at the time of stroke (28). Approximately 20% of children experience epilepsy after ischemic stroke incidence (29). Relative to children without epilepsy, those that experience seizures generally exhibit more substantial cognitive impairment (30) and a decrease in their overall quality of life (31).

### 3.3. Environmental factors

The quality of the home environment contributes to outcomes in patients with ischemic stroke, suggesting that efforts to support parental and family functioning offer opportunities to optimize children's mental health and social outcomes (32). The impact of environmental factors (socioeconomic status and quality of life) on cognitive abilities (expressive and receptive language, adaptive abilities, and social abilities) increased over time after childhood stroke and even exceeded the impact of impairment factors (33). The relationship between socioeconomic status and pediatric health has been well-documented over many years (34). One study found that socioeconomic status was a better predictor of cognitive outcome in childhood arterial ischemic stroke than clinical factors (35). For example, the financial situation of the family may affect the quantity and quality of treatment, and parental education may be linked to children's cognitive reserve. Therefore, future pediatric studies on the prediction of cognitive function should effectively control participants' socioeconomic status. Most importantly, we need to pay more attention to the treatment of children with low socioeconomic status, such as providing more funding and resources.

The above reports clearly emphasize that age, pathological lesion characteristics, and epilepsy co-occurrence can all affect cognitive outcomes in children following ischemic stroke. Differences in reported findings among studies may be attributable to differences in experimental design (cross-sectional vs. longitudinal studies, variations in patient age, and/or differences in disease course) or the specific characteristics of brain development or plasticity in particular patient populations (15).

## 4. Treatment of cognitive impairment

Few studies to date have reported on the rehabilitation of cognitive impairment in children following brain injury, and the underlying evidence is thus limited, with research specifically focusing on post-stroke outcomes in this population being even less common. Non-pharmacological treatments for cognitive impairment that have been explored to date mainly include exercise training, psychological intervention, neuromodulation strategies, computer-assisted cognitive training, brain-computer interfaces (BCI), virtual reality (VR), music therapy, and acupuncture. The goal of rehabilitative strategies in children following brain injury is to allow children to return to their homes and schools as quickly as possible.

### 4.1. Physical exercise

Physical exercise has been explored as a promising neuroprotective and anti-ischemic intervention for patients with ischemic stroke (adults) and animals (36–38), with some evidence suggesting that it can regulate excitatory signal transduction to preserve neurological function (39). Exercise can also boost cerebrovascular efficacy, potentially reducing infarct size and increasing the number of viable cells surrounding the infarcted lesion (36). Such preventative physical activity can also preserve synaptic plasticity in the context of ischemia, and specific therapeutic approaches have been explored as a means of promoting plasticity and improving overall cognitive function (39). Ischemic preconditioning is an interventional approach that has been shown to be effective in individuals suffering from transient non-fatal ischemic periods, conferring adaptive intracellular changes to neuronal electrophysiological properties that can improve the ability of tissues to tolerate future ischemic events (40).

Long-term exercise training after ischemia has been found to enhance the induction of learning-dependent long-term potentiation (LTP) in the CA3 area of the hippocampus (41). Short-term moderate-intensity treadmill exercise was also shown to improve hippocampus-dependent episodic fear memory and other cognitive functions in two rat models of ischemic brain injury (42). Further evidence suggests that exercise can enhance short-term plasticity by improving paired-pulse facilitation (PPF), which promotes the coding of situational and spatiotemporal information by enhancing hippocampal nerve regeneration and facilitating neuronal circuit reorganization (43).

In adults, some studies have found that physical exercise had a positive effect on the global cognitive functioning of patients with stroke (44–47). Wang et al. showed that the combination of physical exercise and cognitive training was more efficacious than cognitive training alone as a means of improving cognitive impairment after stroke in adults (48). Moriya et al. established that moderate-intensity aerobic exercise enhances prefrontal cortex activity and improves working memory performance in patients with post-stroke as assessed by near-infrared spectroscopy (49), while Cotman et al. observed that aerobic exercise benefits cognition, likely through the upregulation of growth factors including BDNF, IGF-1, and VEGF, thus promoting neurogenesis and angiogenesis, particularly in the hippocampus (50).

In children, from a population perspective, moderate-to-vigorous physical activity, especially vigorous physical activity (51), is associated with improved cognitive function in normal prepubertal children (51, 52), as well as in children with ADHD (53) and cerebral palsy (54). Both long-term intervention (>6 months) (51, 52) and short-term intervention (7 days) (55) increased the hippocampal gray matter volume significantly. There are also significant changes in the EEG theta and alpha band power spectra immediately after intervention (51). From the perspective of an interventional approach, different types of physical activity are thought to differentially activate children's brains either through physiological mechanisms or by activating similar brain regions during physical and cognitive tasks; specific or standardized programs are, however, lacking. There are also studies suggesting that not every child benefits from interventions in the same way and that individual differences vary widely (56). As physical fitness comprises both muscle and neuromuscular components, some researchers believe that physical fitness represents a better outcome predictor than physical activity (57).

Exercise training triggers several complex processes that can interact to protect and preserve neuronal function following ischemic injury (38), ameliorating cognitive recovery by improving synaptic plasticity and promoting new neuronal circuit reorganization. Physical exercise, thus, holds great promise as an interventional approach for treating cognitive impairment following ischemic stroke in children, although further research is necessary to understand the extent to which these preclinical findings are applicable to children with ischemic stroke.

### 4.2. Psychological interventions

Psychological interventions are critical means of treating cognitive impairment following ischemic stroke in children, offering guidance regarding available resources and rehabilitative strategies that can help children return to school. Such interventions are broadly divided into strategy training and cognitive retraining approaches, with some studies suggesting that strategy training is the more efficacious of the two (58).

Strategy training is the most popular psychological intervention used at present. While children present with specific cognitive deficits following ischemic stroke, their cognitive advantages can be leveraged to overcome these deficits in particular environments (59). Evidence on the utility of strategy training for the treatment of cognitive impairment is designated as NHMRC grade D, consistent with very low-grade evidence (58), although it currently remains the only recommended treatment supported by direct medical evidence. Successful implementation is dependent upon a comprehensive neuropsychological assessment of the child's cognitive deficits and advantages, as well as an understanding of their individual environmental needs. Effective communication among health professionals, families, and schools is also critical to ensure that children are placed in a supportive environment that provides them with the best possible developmental opportunities (59). One meta-analysis of patients with sickle-cell disease (SCD)-related infarct found that those undergoing psychoeducational interventions including cognitive behavioral therapy, particularly in family settings, showed positive outcomes (60). Three studies have reported on the training of working memory and memory strategy as a means of improving cognitive function in children after stroke (61–63). It has been found that tutoring combined with memory training was more effective than individual tutoring alone and was linked to more positive outcomes (61, 62).

Cognitive retraining has been a focus of increasing research interest in recent years. This strategy primarily relies on an assessment of the degree of cognitive impairment followed by training according to their specific cognitive abilities. A randomized controlled study of children with central nervous system injuries found that a cognitive remediation program (CRP) improved both attention and academic performance (64). Recla et al. showed that a 1-month intensive memory-focused training program (IM-FTP) improved children's ability to learn semantically related and irrelevant words, while also improving their immediate prose memory (65). Functional magnetic resonance imaging (fMRI) analyses of these children revealed that the IM-FTP treatment was associated with functional changes in the left lower frontal cortex. The left lower frontal gyrus is closely associated with the left posterior middle temporal gyrus, which plays a vital role in syntactic analysis (66) and vocabulary selection (67), which is why this is an area that is stimulated during intensive memory training (65).

Through the Swedish Memory and Attention Re-Training and parental coaching program, van't Hooft et al. similarly determined that cognitive retraining of children was able to enhance attention, memory, social interaction, and parental stress outcomes (68). A meta-analysis found that most studies utilizing remote technology-based training programs reported treatment-related improvements in cognition and behavior. For example, remote computerized cognitive training can improve visual-spatial working memory (69). However, substantial heterogeneity exists among the studies published to date (70).

Due to the heterogeneity of neurological behaviors in children after central nervous system injuries, there has been no universal adaption of specific therapeutic programs. Instead, individualized interventional plans are formulated in accordance with the needs of each child. It is thus essential that schools, families, and rehabilitation teams regularly assess and discuss these plans to ensure that children are provided with appropriate environmental and educational programs capable of fostering their cognitive recovery.

### 4.3. Neuromodulation

Neuromodulation therapy has recently emerged as a promising therapeutic modality capable of remediating cognitive function in the context of cerebral injuries including Parkinson's disease (71) and traumatic brain injury (72). As cerebral oscillation patterns are altered following ischemia, electrical or magnetic stimulation may be able to improve overall neural network function by restoring abnormal electrical activity and plasticity (73). Several non-invasive neuromodulatory approaches have been explored as tools for improving cognitive function in children with ischemic stroke, including transcranial direct current stimulation (tDCS) (74) and repetitive transcranial magnetic stimulation (rTMS) (75). The rTMS approach utilizes a coil to generate a magnetic field capable of penetrating the scalp and inducing changes in excitability through a mechanism similar to LTP-LTD, thereby augmenting neuronal plasticity (76). One meta-analysis found that low-frequency (≤1 Hz) rTMS in the unaffected hemisphere of patients (adults) suffering from post-stroke aphasia could effectively improve overall language function (77). Malone et al. posited that patients with childhood ischemic stroke may benefit from rTMS when appropriate operational parameters are employed (78), while Gillick et al. explored optimal tDCS parameters for use in the treatment of children following ischemic stroke, including current intensity, electrode size, location, and stimulation duration (79). There are also invasive neuromodulatory approaches, such as deep brain stimulation (DBS). DBS necessitates the implantation of a pair of electrodes in the brain parenchyma, with the electrodes connected to a pulse generator implanted in the chest. Much like rTMS, DBS can target specific brain regions, and parameters such as voltage intensity and frequency can be customized according to the patient's condition. Importantly, the transmission of specific electrical activity patterns *via* DBS can influence oscillatory activity (80, 81). Increased levels of brain-derived neurotrophic factor (BDNF), vascular endothelial growth factor (VEGF), and synaptic markers such as synaptophysin were detected within 2.5 h of DBS treatment in rats (82). DBS can also improve overall network function by enhancing synaptic plasticity and normalizing disordered oscillatory activity.

While patients with cognitive impairment after brain injury benefit from neuromodulation therapy, the mechanisms underlying these benefits are poorly understood. The development of novel non-invasive neuromodulatory technologies will offer a convenient, cost-effective, safe, and painless means of facilitating cognitive rehabilitation in children following ischemic stroke. As such, future research should focus on optimizing neuromodulatory treatment strategies by the identification of appropriate biomarkers and therapeutic parameters associated with positive patient outcomes.

### 4.4. Other interventions

Multimodal stimulatory approaches, including auditory, visual, olfactory, and exercise-based stimulation, can enhance neuroplasticity and promote cognitive recovery after stroke. This has been preliminarily confirmed in rat models of traumatic brain injury (83). While the current results are promising, more research is needed to make conclusive statements and successfully apply these methods to daily clinical life. Multidisciplinary collaborations help improve current neurotechnologies and provide guidance for future implementations.

#### 4.4.1. Computer-assisted cognitive training

In recent years, computer-assisted cognitive rehabilitation has been regarded as a good alternative or supplement to traditional cognitive rehabilitation. Computer-assisted cognitive training is beneficial to improve the cognitive ability of patients and restoring the overall functional status of patients. It is widely used in cognitive impairment after stroke in adults (84, 85). However, research regarding its use for cognitive impairment in children has largely focused on psychiatric conditions such as ADHD (86) or autism spectrum disorder (87).

#### 4.4.2. Brain-computer interfaces (BCI)

Brain-computer interfaces-based cognitive training is another emerging area in the neurorehabilitation field; this involves the reception of nerve cell signals, identifying and classifying their activity, and translating them into computer-recognized instructions. In adults, BCI treatment of post-stroke cognitive impairment (PSCI) reportedly results in improvements in executive function (88, 89), attention (90), memory (90–92), language (91), and visuospatial abilities (91, 93). In children, Munoz et al. applied the EEG-BCI system to improve attention ability in patients with ADHD (94). Friedrich et al. introduced a BCI application combining neurofeedback and biofeedback to treat children with autism spectrum disease (95). Kim et al. found that BCI can improve logical thinking, problem-solving, and attention to external stimuli in children with spastic cerebral palsy (96). However, there are no reports on the application of this approach for treating cognitive impairment in children, excepting stroke which included cerebral palsy.

#### 4.4.3. Virtual reality

In the past decade, VR has been widely concerned, and its technological progress has surpassed clinical research. A particular property of VR is that it creates the illusion that a person is interacting with a synthetic world. In children's cognitive rehabilitation therapy, VR is widely used, such as improving happiness, relaxation, and anxiety (97), promoting upper limb recovery after ischemic stroke (98), autism spectrum disorder (99), and intellectual disabilities (100). The application of cognitive impairment in children after stroke has not been reported.

#### 4.4.4. Music therapy

Brain imaging studies have shown that the neural activity associated with listening to music extends far beyond the auditory cortex, involving a wide-spread bilateral network of frontal, temporal, parietal, and subcortical regions related to attention, semantic and musical syntactic processing, memory, and motor function (101, 102). In adults, regular music listening during the subacute phase of stroke promotes recovery of verbal memory and focused attention (103), and fine-grained structural reorganization (as indicated by increased gray matter volume, GMV) in the network of frontolimbic brain regions (104). In children with neurological disorders, music therapy has been found to stabilize vital signs during and after treatment, reflected by reduced heart and respiratory rates and increased oxygen saturation (105). We hypothesize that music therapy during the early stages of recovery from stroke could serve as a valuable supplement to patient care by providing an individualized, easily implemented, and inexpensive means of promoting cognitive recovery.

#### 4.4.5. Acupuncture

Acupuncture has been shown to be a safe potential alternative intervention for the treatment of post-stroke patients with cognitive impairment (106). Its mechanism may mainly improve cognitive function after stroke by promoting synaptic plasticity (107). However, no corresponding studies have been conducted on children to date.

## 5. Limitations and future prospects

There is significant heterogeneity in the available studies of pediatric ischemic stroke patients due to differences in experimental design, evaluation methodology, tested interventions, and stroke subgroups. The cognitive function of children is not comparable across age groups, and as such many stroke-related cognitive deficiencies may only manifest over the course of patient growth and development. As such, larger longitudinal studies are essential to fully understand the relative value of different interventional strategies in this vulnerable patient population. To ensure access to effective personalized treatment, it is also critical that biomarkers of cognitive impairment be identified, particularly if such biomarkers can be evaluated using MRI or EEG data modeling approaches. The mechanisms whereby current treatments may benefit patient cognitive function are also not currently understood, and more basic and clinical research is thus essential to facilitate evidence-based treatment.

## 6. Conclusion

Stroke-related cognitive impairment in children has been a focus of increasing research interest in recent years. Impairment is influenced by patient age, pathological characteristics, combined epilepsy status, and environmental factors. Non-pharmacological treatments for cognitive impairment that have been explored to date primarily include exercise training, psychological intervention, neuromodulation strategies, computer-assisted cognitive training, BCI, VR, music therapy, and acupuncture. Most of these interventions are easily implemented and inexpensive strategies that can promote cognitive recovery. In childhood stroke, the only interventions explored in detail to date are psychological interventions and neuromodulatory strategies. However, evidence regarding the efficacy of these interventions is relatively weak. In future studies, the active application of a range of interventions is warranted to improve pediatric cognitive function, and neuroimaging and electrophysiological measurement techniques should be used to identify biomarkers capable of predicting cognitive impairment, facilitating early diagnosis, guiding treatment, and thereby improving patient prognosis. Larger multi-center prospective longitudinal studies are also required to provide more accurate evidence-based guidance for the treatment of patients with childhood stroke.

## Author contributions

GX contributed to conception and design and drafted manuscript. FH, WZ, and JQ contributed to acquisition and interpretation and drafted manuscript. PZ and QZ critically revised manuscript. All authors contributed to the article and approved the submitted version.
